# Racial inequality in COVID-treatment and in-hospital length of stay in the US over time

**DOI:** 10.3389/fpubh.2022.1074775

**Published:** 2023-01-11

**Authors:** Benjamin M. Althouse, Charlotte Baker, Peter D. Smits, Samuel Gratzl, Ryan H. Lee, Brianna M. Goodwin Cartwright, Michael Simonov, Michael D. Wang, Nicholas L. Stucky

**Affiliations:** ^1^Truveta, Inc., Bellevue, WA, United States; ^2^Information School, University of Washington, Seattle, WA, United States; ^3^Department of Biology, New Mexico State University, Las Cruces, NM, United States; ^4^Yale School of Medicine, New Haven, CT, United States

**Keywords:** COVID-19, SARS-CoV-2, remdesivir, treatment, disparities, health equity, race

## Abstract

**Introduction:**

Demonstrated health inequalities persist in the United States. SARS-CoV-2 (COVID) has been no exception, with access to treatment and hospitalization differing across race or ethnic groups. Here, we aim to assess differences in treatment with remdesivir and hospital length of stay across the four waves of the pandemic.

**Materials and methods:**

Using a subset of the Truveta data, we examine the odds ratio (OR) of in-hospital remdesivir treatment and risk ratio (RR) of in-hospital length of stay between Black or African American (Black) to White patients. We adjusted for confounding factors, such as age, sex, and comorbidity status.

**Results:**

There were statistically significant lower rates of remdesivir treatment and longer in-hospital length of stay comparing Black patients to White patients early in the pandemic (OR for treatment: 0.88, 95% confidence interval [CI]: 0.80, 0.96; RR for length of stay: 1.17, CI: 1.06, 1.21). Rates became close to parity between groups as the pandemic progressed.

**Conclusion:**

While inpatient remdesivir treatment rates increased and length of stay decreased over the beginning course of the pandemic, there are still inequalities in patient care.

## 1. Introduction

Inequalities in medical care in the United States (US) are widespread across socioeconomic (SES) and racial boundaries (1). The SARS-CoV-2 (COVID) pandemic has only intensified these inequalities (1). Zelner et al. (2) found a 5.5-fold increase in incidence and age-standardized mortality in Black or African American patients (Black) as compared with White patients, though their study was limited to the state of Michigan. In a systematic review of COVID outcomes, Mackey et al. (3) showed that Black patients experienced higher rates of COVID infection and overall mortality, but not COVID-related mortality. However, no identified studies examined the rates of COVID treatment or length of inpatient hospital stay (LoS). With ongoing calls for healthcare reform, racial equality, and the end of systemic racism, it is of key importance to identify areas where systematic inequalities have been bettered, where they still exist, and how they have changed over time.

Given the growing availability of effective COVID treatments over the course of the pandemic (4) and the severity of unequal distribution of these treatments between racial groups, we aimed to assess the differences in treatment with remdesivir, as well as LoS between Black and White patients using one of the most complete, timely, and highest quality aggregation of electronic health records (EHR): the Truveta, Inc., data platform.

In May 2020, the US Food and Drug Administration (FDA) issued an emergency use authorization (5) for the use of remdesivir in hospitalized patients with severe disease due to COVID-19 because of its demonstrated effectiveness in reducing LoS in hospitalized patients (6, 7). Remdesivir was the first antiviral given emergency use authorization by the FDA. It is an adenosine nucleoside triphosphate analog that interferes with viral RNA-dependent RNA polymerase (8). The FDA subsequently approved the use of remdesivir in hospitalized patients (October 2020) and then in non-hospitalized patients who are at high risk for progression to severe COVID-19 (typically defined as having a high-risk comorbid medical condition) (5). It has been shown to significantly reduce COVID-related severity and duration of illness (9). We focused on remdesivir because it is one of the most common COVID treatments with emergency use authorization being granted early in the pandemic and treatment rates are well represented in the Truveta dataset.

Electronic health record (EHR) data provide a comprehensive view of a patient's journey through COVID prophylaxis, infection, symptomology, treatment, and ultimate outcome. Recent advances in data science, data security technology, and ethical and legal reviews of data storage, processing, and distribution have allowed unprecedented access to such data stores. For this study, we aimed to examine the changes in the delivery of COVID treatments and in-hospital LoS for COVID by race.

We aim to assess racial and ethnic inequities in treatment with remdesivir and hospital length of stay across the four waves of the pandemic after controlling for confounding factors, such as comorbidities, age, and sex.

## 2. Materials and methods

### 2.1. Data source and study population

Our study included all inpatient COVID patients present in the de-identified Truveta electronic health records. Truveta provided the de-identified medical records used in this study on 22 June 2022. Truveta is a collective of healthcare systems that came together to aggregate EHR data with the purpose of enabling research. Currently, this collective includes 24 members who provide patient care in over 20,000 clinics and 700 hospitals across 43 states. We provide a comprehensive datasheet on the national population represented Truveta data and every population being studied. These datasheets include patient counts, diversity, completeness, and timeliness statistics, as well as the sources of all data. Truveta Studio includes more than 72 million de-identified patient journeys available for the study. For this paper, we studied a subset of the Truveta patient population. Based on the evaluation of this information, we believe that the study population is representative of the general population. While we do not have access to high-level geographic data for this study, the focus on large healthcare systems might create a slight bias toward urban settings. Updated data are provided daily to Truveta. Through syntactic normalization, similar data fields from different systems are mapped to a common schema referred to as the Truveta Data Model (TDM). Once organized into common fields, the values are normalized to common ontologies, such as ICD-10, SNOMED-CT, LOINC, RxNorm, and CVX, through semantic normalization. The normalization process employs an expert-led, artificial intelligence-driven process to accomplish high-confidence modeling at scale. The data are then de-identified by an expert determination under the HIPAA Privacy Rule.

The data lag in the Truveta data is 7–14 days. There can be delays in clinician documentation (e.g., it may take several days following a discharge for a provider to record a discharge event). In addition, while we receive data daily from our health systems, the normalization, de-identification, and quality check processes require 7–14 days to complete to ensure the highest quality data are available in Truveta Studio. Once de-identified, the data are available for analysis in R or python using Truveta Studio. The Providence Health Care Institutional Review Board has declared this study not human research (STUDY2022000435).

### 2.2. Inclusion and exclusion criteria

Our study period spanned from 1 March 2020 to 1 March 2022. Inclusion criteria included COVID-19 diagnosis, either from a laboratory result or an assigned diagnosis, during one of our treatment windows. Patients were excluded if their records were missing sex and age information. For those patients who were missing encounter times and for those patients who received remdesivir, we excluded those patients missing drug administration times. The data provided by healthcare systems were comprised of patients from 40 US states.

### 2.3. Data analysis

We compared three remdesivir treatment windows corresponding to waves of the pandemic: “December 2020” from 1 December 2020 to 28 February 2021; “Delta” from 1 June 2021 to 31 August 2021; and “Omicron” from 1 December 2021 to 28 February 2022 (10). As remdesivir was given emergency authorization by the FDA on 1 May 2020, we included an additional wave—“wild type” from 1 March 2020 to 31 May 2020 in the LoS analysis.

Our main comparisons were between remdesivir treatment between Black patients and White patients as well as LoS in these two groups. Logistic regression with remdesivir treatment (yes/no) as the model outcome was used to estimate differences between racial groups adjusting for age and sex, as well as the following comorbidities: diabetes, hypertension, chronic kidney disease (CKD), liver disease, immunocompromised state, and cancer (ICD-10 and SNOMED codes are provided in the Supplementary material). Specifically, we obtained the odds ratio from the logistic regressions. Poisson and Negative Binomial generalized linear regression models were fit to counts of days of LoS and compared with the Akaike information criterion (AIC) (11), adjusting for the same covariates. We note that this is exploratory and does not account for censoring in days of LoS, as a full survival analysis would. This is due to a de-identification process which removes elements that introduce risk of re-identification. The Kolmogorov–Smirnov test was used to test for differences in ages between Black patients and White patients.

All analyses were conducted in R software version 4.1.3 (12), using packages dplyr (13), ggplot2 (14), gtools (15), MASS (16), xtable (17), bit64 (18), lmtest (19), and scales (20).

## 3. Results

**Figure 1:**
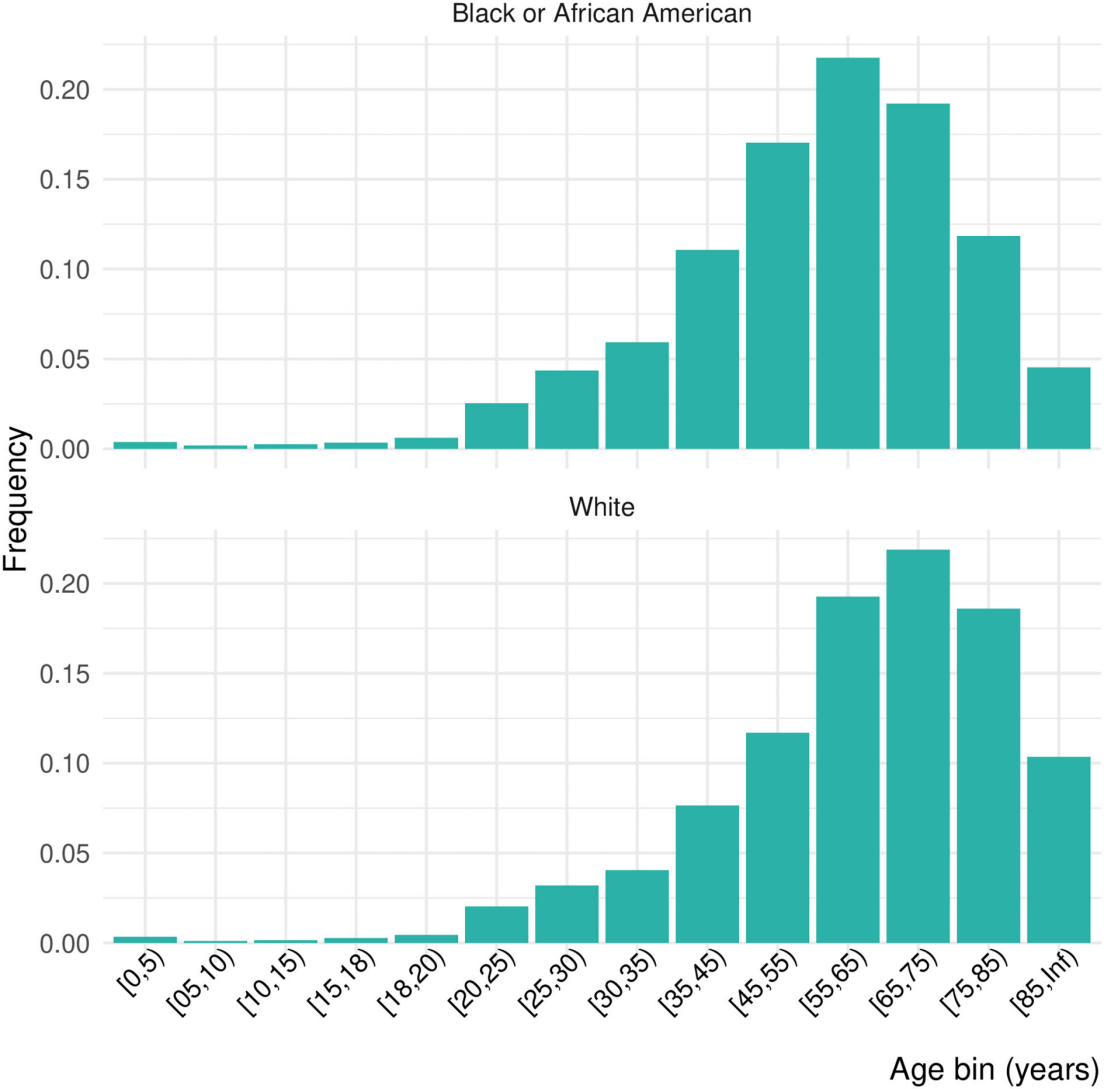
Distribution of ages by age bin and race.

**Figure 2:**
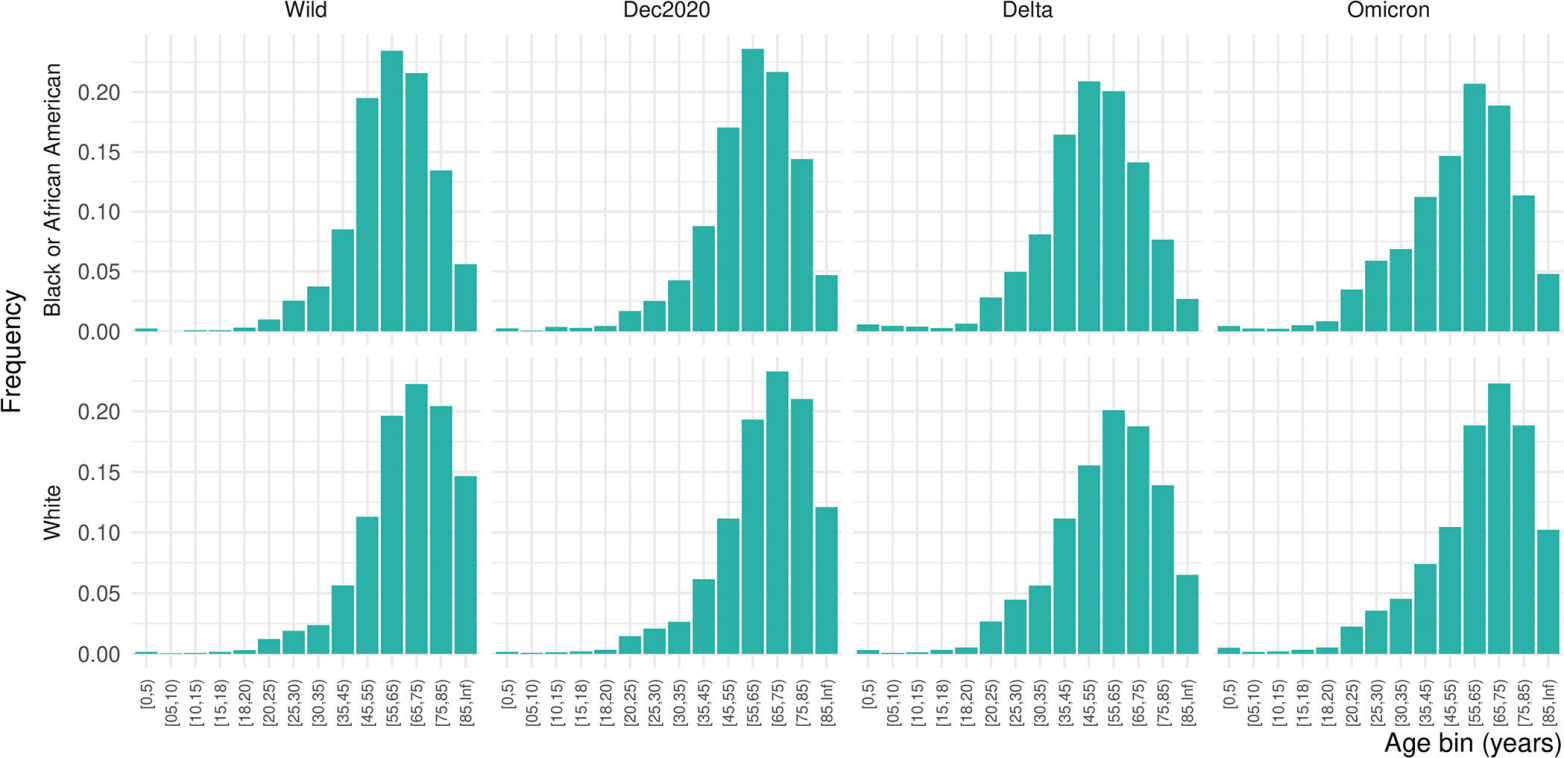
Distribution of ages by age bin, race, and pandemic wave.

Of 556,134 patients who had a COVID diagnosis or laboratory test during one of the four pandemic periods of interest, 10.6% were inpatients, giving a final study population of 64,650 (Table 1). Females made up 51.2% of the inpatient sample, 14.6% Black and 70.1% White. Due to the small proportion of Asian, American Indian or Alaska Native, Native Hawaiian or other Pacific Islander, or patients missing data on race, we focus only on Black and White patients in these results. Nearly 46% of the population was over 65 years of age. The distribution of ages was significantly shifted to younger ages for Black patients than White patients (Kolmogorov–Smirnov test, D^+^ = 0.159, *p* < 0.001; Figure 1). This persisted across waves of the pandemic (Figure 2). Median age of hospitalizations for Black patients was 58.7 years (25–75% quantile: 44.5–69.9) compared with 65.4 years (25–75% quantile: 51.5–76.9) for White patients. We obtained the percentage of other prevalent comorbidities (diabetes, hypertension, CKD, liver disease, immunocompromised state, and cancer) for both groups and stratified by the wave (Table 2).

**Table 1:** Demographic characteristics of the population affected by each wave of COVID-19 and their comorbidities (the age distributions are given in Figure 2).

	**Wild**	**December 2020**	**Delta**	**Omicron**	**Overall**
	**(*N* = 5,803)**	**(*N* = 17,824)**	**(*N* = 12,287)**	**(*N* = 28,736)**	**(*N* = 64,650)**
Sex					
Female	2,840 (48.9%)	8,781 (49.3%)	6,355 (51.7%)	15,094 (52.5%)	33,070 (51.2%)
Male	2,963 (51.1%)	9,043 (50.7%)	5,932 (48.3%)	13,642 (47.5%)	31,580 (48.8%)
Race					
American Indian or Alaska	27 (0.5%)	102 (0.6%)	107 (0.9%)	229 (0.8%)	465 (0.7%)
Native					
Asian	177 (3.1%)	522 (2.9%)	244 (2.0%)	615 (2.1%)	1,558 (2.4%)
Black or African American	1,339 (23.1%)	2,537 (14.2%)	1,594 (13.0%)	3,991 (13.9%)	9,461 (14.6%)
Native Hawaiian or Other Pacific	18 (0.3%)	64 (0.4%)	68 (0.6%)	112 (0.4%)	262 (0.4%)
Islander					
White	3393 (58.5%)	12,553 (70.4%)	8,789 (71.5%)	20,602 (71.7%)	45,337 (70.1%)
Missing	849 (14.6%)	2,046 (11.5%)	1,485 (12.1%)	3,187 (11.1%)	7,567 (11.7%)
Ethnicity					
Hispanic or Latino	889 (15.3%)	2,985 (16.7%)	1,994 (16.2%)	3,536 (12.3%)	9,404 (14.5%)
Not Hispanic or Latino	4,670 (80.5%)	14,350 (80.5%)	9,897 (80.5%)	24,250 (84.4%)	53,167 (82.2%)
Missing	244 (4.2%)	489 (2.7%)	396 (3.2%)	950 (3.3%)	2,079 (3.2%)
Age bracket					
[0,5)	12 (0.2%)	42 (0.2%)	55 (0.4%)	209 (0.7%)	318 (0.5%)
[05,10)	3 (0.1%)	18 (0.1%)	25 (0.2%)	63 (0.2%)	109 (0.2%)
[10,15)	3 (0.1%)	30 (0.2%)	30 (0.2%)	68 (0.2%)	131 (0.2%)
[15,18)	7 (0.1%)	44 (0.2%)	47 (0.4%)	124 (0.4%)	222 (0.3%)
[18,20)	22 (0.4%)	69 (0.4%)	82 (0.7%)	186 (0.6%)	359 (0.6%)
[20,25)	79 (1.4%)	316 (1.8%)	379 (3.1%)	825 (2.9%)	1,599 (2.5%)
[25,30)	137 (2.4%)	451 (2.5%)	605 (4.9%)	1,296 (4.5%)	2,489 (3.9%)
[30,35)	181 (3.1%)	589 (3.3%)	783 (6.4%)	1,588 (5.5%)	3,141 (4.9%)
[35,45)	452 (7.8%)	1,340 (7.5%)	1,597 (13.0%)	2,508 (8.7%)	5,897 (9.1%)
[45,55)	831 (14.3%)	2,272 (12.7%)	2,043 (16.6%)	3,259 (11.3%)	8,405 (13.0%)
[55,65)	1,218 (21.0%)	3,577 (20.1%)	2,403 (19.6%)	5,368 (18.7%)	12,566 (19.4%)
[65,75)	1,241 (21.4%)	4,023 (22.6%)	2,097 (17.1%)	5,952 (20.7%)	13,313 (20.6%)
[75,85)	996 (17.2%)	3,292 (18.5%)	1,467 (11.9%)	4,769 (16.6%)	10,524 (16.3%)
[85,Inf)	621 (10.7%)	1,761 (9.9%)	674 (5.5%)	2,521 (8.8%)	5,577 (8.6%)
Treated with remdesivir					
Yes	222 (3.8%)	6,948 (39.0%)	4,716 (38.4%)	4,833 (16.8%)	16,719 (25.9%)
No	5,581 (96.2%)	10,876 (61.0%)	7,571 (61.6%)	23,903 (83.2%)	47,931 (74.1%)
Cancer					
Yes	318 (5.5%)	965 (5.4%)	626 (5.1%)	1,870 (6.5%)	3,779 (5.8%)
No	5,485 (94.5%)	16,859 (94.6%)	11,661 (94.9%)	26,866 (93.5%)	60,871 (94.2%)
Chronic kidney disease (CKD)					
Yes	379 (6.5%)	1,432 (8.0%)	738 (6.0%)	2,197 (7.6%)	4,746 (7.3%)
No	5,424 (93.5%)	16,392 (92.0%)	11,549 (94.0%)	26,539 (92.4%)	59,904 (92.7%)
Diabetes					
Yes	1,033 (17.8%)	3,353 (18.8%)	1,931 (15.7%)	4,774 (16.6%)	11,091 (17.2%)
No	4,770 (82.2%)	14,471 (81.2%)	10,356 (84.3%)	23,962 (83.4%)	53,559 (82.8%)
Hypertension					
Yes	2,113 (36.4%)	6,617 (37.1%)	3,941 (32.1%)	10,382 (36.1%)	23,053 (35.7%)
No	3,690 (63.6%)	11,207 (62.9%)	8,346 (67.9%)	18,354 (63.9%)	41,597 (64.3%)
Liver disease					
Yes	219 (3.8%)	668 (3.7%)	564 (4.6%)	1,557 (5.4%)	3,008 (4.7%)
No	5,584 (96.2%)	17,156 (96.3%)	11,723 (95.4%)	27,179 (94.6%)	61,642 (95.3%)
Immunocompromised					
Yes	405 (7.0%)	1,208 (6.8%)	831 (6.8%)	2,468 (8.6%)	4,912 (7.6%)
No	5,398 (93.0%)	16,616 (93.2%)	11,456 (93.2%)	26,268 (91.4%)	59,738 (92.4%)

**Table 2:** Percentages of each population (Black patients and White patients) for each comorbidity (the age distributions are given in Figure 2) by COVID wave.

**Race**	**Comorbidity**	**Wild**	**December 2020**	**Delta**	**Omicron**	**Overall**
Black or African American	Cancer	52 (3.88%)	115 (4.53%)	56 (3.51%)	195 (4.89%)	418 (4.42%)
White	Cancer	235 (6.93%)	774 (6.17%)	502 (5.71%)	1,486 (7.21%)	2,997 (6.61%)
Black or African American	CKD	107 (7.99%)	328 (12.93%)	132 (8.28%)	412 (10.32%)	979 (10.35%)
White	CKD	217 (6.4%)	974 (7.76%)	504 (5.73%)	1,565 (7.6%)	3,260 (7.19%)
Black or African American	Diabetes	273 (20.39%)	603 (23.77%)	281 (17.63%)	772 (19.34%)	1,929 (20.39%)
White	Diabetes	566 (16.68%)	2,251 (17.93%)	1,340 (15.25%)	3,342 (16.22%)	7,499 (16.54%)
Black or African American	Hypertension	541 (40.4%)	1,069 (42.14%)	575 (36.07%)	1,574 (39.44%)	3,759 (39.73%)
White	Hypertension	1,283 (37.81%)	4,774 (38.03%)	2,887 (32.85%)	7,697 (37.36%)	16,641 (36.71%)
Black or African American	Immunocompromised	79 (5.9%)	158 (6.23%)	96 (6.02%)	313 (7.84%)	646 (6.83%)
White	Immunocompromised	285 (8.4%)	936 (7.46%)	636 (7.24%)	1,876 (9.11%)	3,733 (8.23%)
Black or African American	Liver disease	34 (2.54%)	74 (2.92%)	65 (4.08%)	161 (4.03%)	334 (3.53%)
White	Liver disease	138 (4.07%)	474 (3.78%)	405 (4.61%)	1,165 (5.65%)	2,182 (4.81%)

**Figure 3:**
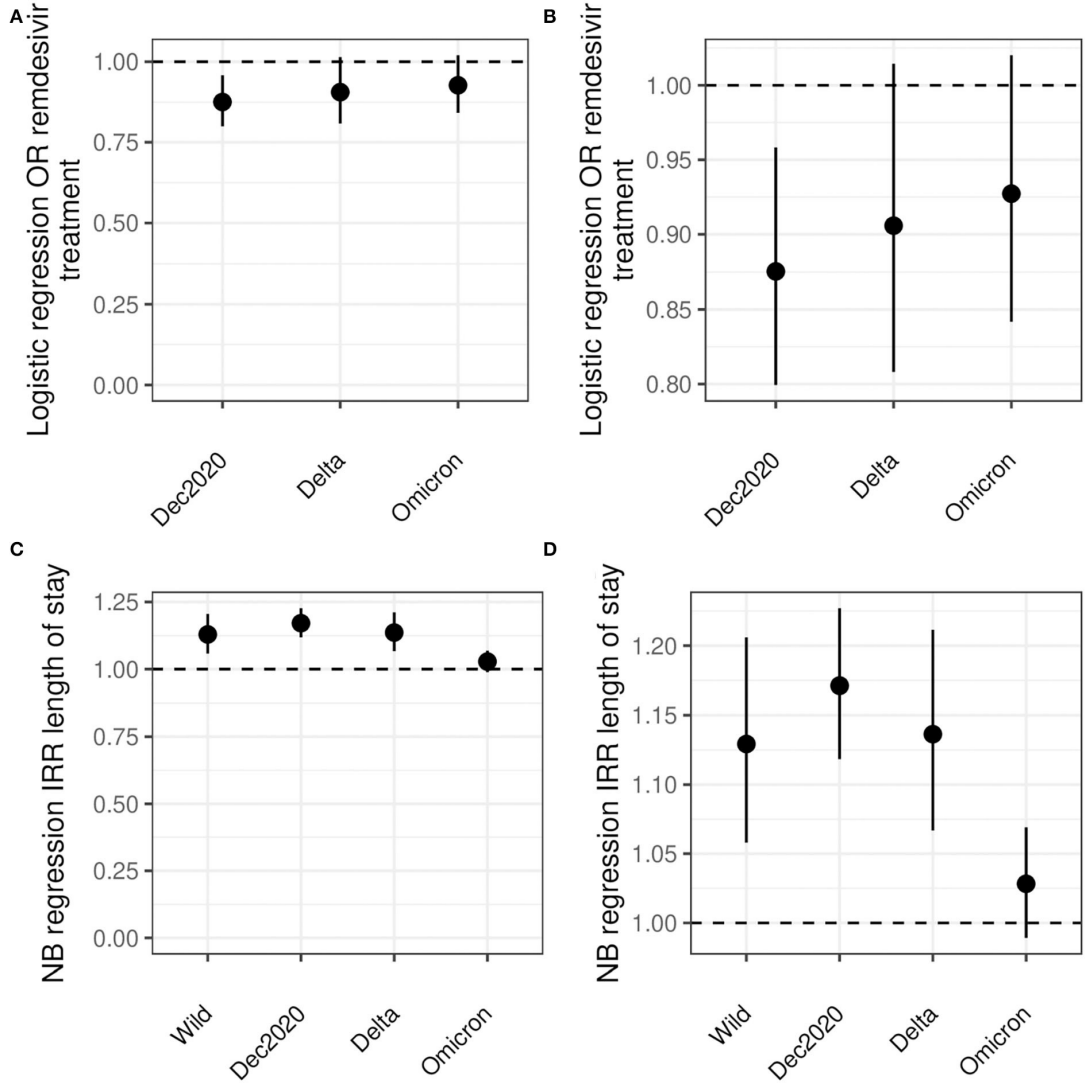
Logistic and NB regression results. **(A, B)** show the odds ratio for remdesivir treatment comparing Black and White inpatients. **(C, D)** show the risk ratio for LoS comparing Black and White inpatients. All analyses are adjusted for age, sex, diabetes, hypertension, CKD, liver disease, immunocompromised state, and cancer. Note the differences in y-axes between **(A, C)** and between **(B, D)**.

Including adjustment for diabetes, hypertension, CKD, liver disease, immunocompromised state, and cancer, Black patients had significantly lower odds to receive remdesivir compared with White patients (OR 0.88, CI: 0.80, 0.96) (Figure 3 and Table 3). No statistical difference in receipt of remdesivir was seen for Black patients compared with White patients during the Delta or Omicron waves (9% lower and 7% lower odds, respectively).

**Table 3:** Odds ratio and 95% confidence interval for remdesivir treatment while hospitalized for COVID comparing Black and White inpatients.

**Wave**	**Population**	**Estimate**	**2.5%**	**97.5%**
Dec2020	Black or African American	0.88	0.80	0.96
Delta	Black or African American	0.91	0.81	1.01
Omicron	Black or African American	0.93	0.84	1.02

Adjusted for age, sex, diabetes, hypertension, CKD, liver disease, immunocompromised state, and cancer.

Negative binomial models for days of LoS were preferred over Poisson models by AIC (Supplementary material). After adjustment for age, sex, diabetes, hypertension, CKD, liver disease, immunocompromised state, and cancer, Black inpatients had 5, 16, and 18% longer LoS for the wild type, December 2020, and Delta waves, respectively. There was no statistically significant difference for the Omicron wave (3%) (Table 4).

**Table 4:** Table shows the risk ratio of length of stay (LoS) for Black inpatients as compared with White inpatients.

**Wave**	**Population**	**Estimate**	**2.5%**	**97.5%**
Wild	Black or African American	1.13	1.06	1.21
Dec2020	Black or African American	1.17	1.12	1.23
Delta	Black or African American	1.14	1.07	1.21
Omicron	Black or African American	1.03	0.99	1.07

Adjusted for age, sex, diabetes, hypertension, CKD, liver disease, immunocompromised state, and cancer.

## 4. Discussion

Nearly all aspects of healthcare in our society have been affected disproportionately across SES, geography, and race or ethnic groups. Unfortunately, the COVID pandemic has seen the same pattern (1). Inequalities in access to healthcare, timeliness of treatment delivery, and treatment outcomes have been known for decades (21–26). Our ongoing accumulation of knowledge on COVID outcomes, epidemiology, and treatments allows us to better situate disparities across racial groups.

Here, we find that early in the pandemic, there were broad differences in LoS and remdesivir treatments comparing Black patients and White patients with comorbidities. Hearteningly, our sample of over 60 thousand COVID inpatients shows this gap may be closing and approaching parity between the two groups. We suspect that differences in LoS are in part driven by social and structural determinants such as trust in public health and healthcare, past experiences with clinicians, and variability in care due to provider bias. This would lead to a delay in care, poorer care, and require extra measures to achieve positive patient outcomes. This finding is different to the a study from the Centers for Disease Control and Prevention. In that study, using COVID-Net, a surveillance system with data from 99 counties in 14 states, there was no significant difference in LoS between White and Black patients from 1 March 2020 to 28 February 2021 (27). However, that study did find that non-White patients were much more likely to need to be hospitalized, treated in the intensive care unit, or die from COVID-19 (27). The Black patients were 2.9 times more likely than White patients to be hospitalized and, more importantly here, 3.2 times more likely to be treated in the intensive care unit, a factor that certainly leads to a longer LoS (27). The present study examined the effect modification of the wave of the pandemic on LoS, an analysis not done in the CDC study, so the comparison is limited. Given the emergency use authorization for remdesivir initially was targeted at hospitalized patients and the CDC's findings that Black patients were much more likely to have been hospitalized, we would expect that early on Black patients would have received remdesivir at least as often as their White counterparts. However, we saw the reverse, which underscores the importance of systematic and structural determinants in patient treatment.

Future work to see if similar findings are in data from COVID-Net and analysis including a range of social determinants is needed.

We examined the rates of comorbidities between Black and White patients in our sample and found statistical differences for diabetes hypertension, CKD, liver disease, immunocompromised state, and cancer between race or ethnic groups that attenuated over time (Table 1). In addition, we saw marked differences in the distribution of ages between the groups who were hospitalized; Black patients were hospitalized at ages nearly 10 years younger than White patients and an overall left shift in the age distribution for Black patients. This may indicate the earlier occurrence of the comorbidities in Black populations than White populations. This has been seen for diabetes (28), hypertension (29, 30), CKD (31), liver disease (32), and cancer (33, 34). Remdesivir treatment is not recommended for patients with an eGFR of less than 30 mL/min (equivalent to Stage 4–Severe CKD or Stage 5–End Stage CKD) (35). Black individuals are four times more likely to develop CKD than their White counterparts primarily due to higher rates of hypertension and diabetes (36). In addition, younger patients with CKD of all races and ethnic groups are less likely to see a nephrologist for care compared with their older counterparts, particularly patients who are Black, Hispanic, or of low socioeconomic status (37). The differences seen here could also be due to other behavioral and social factors, such as smoking status, proximity to a food desert or food swamp, access to primary care, unequal access to clinical care including hours of availability, patient refusal for accessing care or receiving specific treatment, employment type, religious or personal beliefs, provider knowledge, bias, or beliefs, or patient's perceived agency to contribute to care decisions. Future work should examine in more detail the drivers of this age discrepancy and interventions implemented to reach younger Black individuals.

Geographic differences in susceptibility to COVID and treatment practice may also contribute to the disparities seen on a national scale. For example, one study showed that environments with high particulate matter were positively correlated with higher mortality in COVID (38). COVID incidence peaked in different geographies at different times. Thus, further study of differences across geography with respect to race or ethnic groups is warranted. A study of the Fangcang Shelter in Wuhan, China, found that patients with fever before admission, patients with a short duration between symptom onset and admission, and patients diagnosed with bilateral pneumonia had a longer LoS than patients without these factors (39). Most patients did not receive western treatments including intravenous medications such as remdesivir. However, unlike the present study, researchers 1) could only study non-severe cases of COVID-19 because the Wuhan triage system assigned only those cases to Fangcang and 2) did not find a statistical difference in LoS between patients with diabetes and without diabetes after adjustment for other factors (39). We did not explicitly incorporate geography or cultural differences into our analyses and leave that for future work.

### 4.1. Strengths and limitations

This study has several specific strengths. Our study is a large study of over 60 thousand patients hospitalized with COVID. Because we used the EHR data, we were able to go beyond ICD-10-CM codes and incorporate laboratory results and SNOMED and RxNorm codes to best identify COVID-19, comorbidities, and medications. In contrast, a 2020 study on COVID-19 mortality from Cleveland Clinic in the US identified COVID-19 solely by a positive PCR test and used data from 495 patients in the first 3 months of the pandemic (40). We benefited from a longer study period and were able to examine multiple waves of the pandemic, not just the period in which the wild type was predominant. This extra time allowed more comprehensive statistical analysis to examine the relationship between factors contributing to a longer LoS and the ability to study the use of remdesivir, which did not become available until later in the pandemic. Even as an exploratory study, these results provide evidence supporting treatment inequalities in the American medical system.

Like all studies, ours is not without limitations. First, we compared only Black and White racial groups of patients for two reasons: 1) while the Truveta platform has representative data on race or ethnic groups, there are still sample size limitations to race or ethnic groups other than Black and White patients; 2) there have been multiple studies examining various aspects in inequalities between Black and White groups, but few or none using data as large and comprehensive as Truveta, and many are from early in the pandemic (1, 3, 40–45). Second, our estimates of differences in LoS between race or ethnic groups are calculated using Poisson and NB regression techniques, which do not account for right- or left-censoring of LoS. Future work could use a survival analysis approach to adjust for censoring.

Third, we studied waves of the pandemic corresponding to the dominant COVID strain at that time, and we do not include information on whether the patient had that dominant strain at the time. That said, the intent of the study was to characterize differences in remdesivir treatment (chosen because of remdesivir's early emergency use authorization and for having a large enough sample size in our data) and not the specific infecting strain type. Fourth, we did not examine the potential effects of limitations in hospital capacity or specifics of healthcare access. Important aspects of access that may have exacerbated disparities in treatment include having a primary care clinician, discrepancies in work or school hours and available hours of an emergency or urgent care, geographic proximity to a medical facility, or access to a facility that accepted all patients for testing or treatment. Future work should address geographic differences in disparities, local hospital capacity, and all aspects of access to care.

Fifth, the data cannot assess the time to diagnosis of COVID and the severity at admittance. We did not include some factors influencing LoS such as the use of ventilators. Future studies should account for the full spectrum of social determinants of health that drive an individual to seek or avoid healthcare or increase severity at admittance. Sixth, the use of EHR for research always has some data lag for certain aspects of the patient story such as the assignment of a specific ICD code or the finalization of coding for a patient with a complex case. However, while Truveta receives data daily from our health systems, the normalization, de-identification, and quality check processes require 7–14 days lag.

Finally, the inclusion criteria for this study included individuals who had been hospitalized with COVID infection, thus we cannot distinguish those admitted *for* COVID or those admitted for *something else* and COVID was an ancillary diagnosis. As patients were likely tested for COVID upon entering the hospital, there is likely an overestimation of people hospitalized for COVID because the number would include patients there for other reasons. Had we been able to distinguish between these populations, we would have been able to determine if those who were admitted for COVID had a different probability of being administered remdesivir compared with those who were admitted for other reasons. We could then elucidate whether the reason for admission modified the relationship between LoS or COVID-treatment and racial inequality. Future work should include this as a focus.

## 5. Conclusion

This is a large study of over 60 thousand patients hospitalized with COVID and shows differences in treatments and LoS between Black patients compared with White patients before and after adjustment of factors including age, diabetes, CKD, and hypertension. There are still many steps to be taken to achieve equity in healthcare including with COVID-19. Policymakers should prioritize continuing to understand where disparities exist and focusing on modifiable factors that lead to achieving equity in access to healthcare and quality of care.

## Data availability statement

The data analyzed in this study is subject to the following licenses/restrictions: Code necessary to generate all analyses and figures is included in a GitHub repository https://github.com/Truveta/althouse_et_al_covid_equity. Data is available only to Truveta subscribers. Requests to access these datasets should be directed to Truveta Inc., info@truveta.com.

## Author contributions

BA, NS, SG, PS, and BG contributed to conception and design of the study. RL, MW, and MS organized the database. BA, PS, and SG performed the statistical analysis. BA wrote the first draft of the manuscript. CB, NS, BG, and PS wrote sections of the manuscript. All authors contributed to manuscript revision, read, and approved the submitted version.

## Supplementary material

The supplement contains ICD-10, SNOMED, and RxNorm codes which are used to define the outcomes and comorbidities as well as the AIC values from the Poisson regression.
