# Supplementary material for: Racial inequality in COVID-treatment and in-hospital length of stay in the US over time
Source: Front Public Health. 2023 Jan 11;10:1074775. doi: 10.3389/fpubh.2022.1074775 (PMC9876573; doi:10.3389/fpubh.2022.1074775)
Supplement: Supplementary file 1 [file Data_Sheet_1.PDF]

Supplementary Material

DATA ELEMENT DEFINITIONS

Below are the codes used for defining the COVID outcome as well as for the comorbidities.

Remdesivir treatment

RxNorm: 2367757, 2367758, 2284718, 2284957, 2284958, 2284959, 2284960, 2395503, 2395499, 2395500, 2395502, 2395504

COVID Outcome

ICD-10: B97.29, U07.1, Z86.16;

SNOMED CT: 840539006, 870588003, 870589006, 870590002, 870591003, 119731000000000, 119741000000000, 119751000000000, 119981000000000, 1240520000000000, 441590008, 840544004, 1017214008, 1119302008

Comorbidities

Cancer:

ICD-10: C00–C26, C40–C96

CKD:

ICD-10: D63.1, E08.22, E09.22, E13.22, I12, I12.0, I12.9, I13, I13.10, I13.11, I13.2, N18, N18.1, N18.2, N18.3, N18.4, N18.5, N18.6, N18.9, O10.211, O10.212, O10.213, O10.219, O10.22, O10.31, O10.32, O10.33, Q61.2, Q61.3, Q61.8, Z94.0

Diabetes:

ICD-10: E10, E11, E13

Hypertension:

ICD-10: I10, I11, I12, I13, I15

Immunocompromised:

ICD-10: B20, C00–C26, C40–C96, D00–D09, D37–D44, D80–D84, D89, Z94

AIC COMPARISONS OF POISSON AND NEGATIVE BINOMIAL REGRESSIONS

Population	AIC Poisson	AIC NB	Delta AIC
Dec 2020	123955.63	71840.52	-52115.11
Delta	100568.24	51387.44	-49180.80
Omicron	223433.61	125462.91	-97970.70

LOG LIKELIHOOD TEST RESULTS

Wave	Model	Df	Log Likelihood	χ^2 Df	χ^2	Pr($>\chi^2$)
Dec 2020	Poisson	13.00	-61964.81			
Dec 2020	Negative Binomial	14.00	-35906.26	1.00	52117.11	0.00
Delta	Poisson	13.00	-50271.12			
Delta	Negative Binomial	14.00	-25679.72	1.00	49182.80	0.00
Omicron	Poisson	13.00	-111703.81			
Omicron	Negative Binomial	14.00	-62717.46	1.00	97972.70	0.00
